# The influence of perceived landscape value on tourists’ behavioral intentions in historical and cultural blocks

**DOI:** 10.1371/journal.pone.0312491

**Published:** 2025-01-09

**Authors:** Jiaojiao Du, Yilei Wang

**Affiliations:** 1 WeiFang Vocational College, Weifang, China; 2 Huazhong Agricultural University, Hubei, China; Linyi University, CHINA

## Abstract

Historic cultural blocks are areas where a city’s material cultural heritage and humanistic characteristics converge, showcasing the city’s unique features and preserving rich and complete urban memories. Research on historic blocks primarily involves strategies related to protection, renewal, planning, and enhancement. However, there is a paucity of studies that explore the relationship between landscape value perception and tourist behavioral intentions from the perspective of recreation participants during the development and renewal of historic cultural blocks. This study uses the Zhongshan Road and Sifang Road historic cultural blocks in Qingdao as a case example. By collecting multi-source data such as online texts, tourist questionnaires, and interviews, and employing qualitative analysis and structural equation modeling as quantitative analysis methods, the study reveals the structural relationship between landscape value perception, place identity, and tourist behavioral intentions: (1) Landscape value perception has a significant positive effect on tourist behavioral intentions; (2) Landscape value perception has a significant positive effect on place identity; (3) Place identity, as a mediating variable, does not mediate the relationship between ecological landscape value perception and skill education value perception, but partially mediates the relationship between historical cultural value perception and recreational value perception. Based on the research results, feedback on the development effectiveness of historic cultural blocks from the perspective of recreation participants can be visually obtained. This helps identify the pain points in the renewal and development process, pinpoint the perceptual dimensions of landscape value that need improvement, and effectively enhance the multiple dimensions of landscape value perception in the blocks. This, in turn, activates the tourism vitality of historic cultural blocks and forms a virtuous cycle model for their development.

## 1. Introduction

Historical and cultural blocks, as condensed manifestations of urban cultural heritage [1], have emerged as unique tourism resources with individualized city symbols. These areas play a pivotal role in revitalizing and developing tourism, particularly in the post-epidemic era [2]. The concept of historical and cultural blocks, internationally known as "historical areas" [3], has undergone a significant evolution in China. This evolution has progressed through various stages, from "historic traditional blocks" to "historical and cultural protection areas," and finally to the current concept of "historical and cultural blocks" [4]. These are defined as areas rich in cultural relics, with concentrated historical buildings, offering a complete and authentic display of traditional patterns and historical features on a substantial scale [5].

In recent years, cities across China have been undertaking renewal and development projects in these areas, focusing on quality enhancement and quantity reduction. While urban planners and government departments often spearhead these renewal efforts, it is the recreational users who are the primary perceivers of these historical and cultural blocks. This raises a crucial question: How can we perceive the landscape value of historical and cultural blocks from the perspective of recreational users’ participation, and balance the relationship among planners, competent authorities, and users? Addressing this question can provide valuable feedback on the renewal and development of historical and cultural blocks and assess the effectiveness of these initiatives, which forms the core value of this study.

Landscape Perception theory, which originated as an offshoot of environmental psychology [6], provides a foundational framework for understanding how individuals interact with their surroundings. Psychologist Ittelson elucidated landscape perception as the process of receiving and interpreting environmental information. This process involves the environment providing information through various sensory channels, with the perceiver selectively receiving and processing this information. Perception can be further divided into sensation and cognition, where individuals first generate specific feelings about individual attributes of things (such as smell, texture, shape) and then process and organize this sensory information to form an overall understanding. Building on this, Shu Xinyi et al. summarized the process of landscape perception into four steps: landscape stimulation, sensation generation, cognitive sublimation, and emotional response [7].

Landscape value perception, a critical dimension of landscape perception, is inherently multidimensional. Existing studies have endeavored to uncover the connotation of landscape value, using it as a basis for landscape planning and management. Landscape value emerges from the interaction between people and their environment, with individuals ascribing specific meanings and values to landscapes through their perception [8, 9]. In China, research on landscape value initially stemmed from the perspective of heritage conservation, primarily focusing on spaces such as scenic areas [10, 11], historic blocks [12], and ancient villages [13].

Different research fields have expanded the concept of landscape value into multiple dimensions. Wu Meiping (2006) broadened the value system of historic landscapes to encompass historical, artistic, scientific, emotional, social, environmental, and ecological values [14]. Gu Jiang (2009) approached the concept from an economic background, dividing values into emotional and cultural dimensions. The emotional dimension included inheritance, identity, and worship value, while the cultural dimension comprised historical, archaeological, remnant, aesthetic, and educational values. Notably, economic value was analyzed separately [15]. Chen Tongbin (2012) examined the value of West Lake’s cultural landscape in Hangzhou through the lens of Chinese traditional landscape aesthetics, emphasizing its rich historical and cultural deposits and universal value as a world heritage site, while considering six elements of value-bearing [16].

Internationally, the classification of landscape value has been more systematic, with categorizations emerging from various research fields. The Athens Charter of 1933 proposed landscape value types including artistic, historical, and scientific values from the perspective of traditional heritage protection. A significant milestone occurred in 1992 when the 16th World Heritage Committee included cultural landscapes as a subcategory of cultural heritage on the World Heritage List [17]. New Zealand scholars Brown and Reid (2000) developed a comprehensive landscape value typology, organizing it into 13 detailed categories: aesthetic, historical, cultural, spiritual, economic, recreational, biological, learning, life-sustaining, intrinsic, living, future, and healing. Australian economist David Throsby (2001) contributed by adding use and non-use values of heritage from an economic perspective [18]. The 2006 European Landscape Convention (ELC) further expanded the understanding of landscape value by emphasizing the importance of landscape value policy-making and user perceptions. Since then, the composition of landscape value types has continued to evolve with different planning environments [19–23].

However, a notable gap in both domestic and international research is the lack of categorization of landscape value perception types from the perspective of tourists, particularly in the context of historical and cultural blocks. This gap is significant because the emotional connection of tourists to recreational landscape value is reflected in the strength of their behavioral intentions [24].

Tourist behavioral intention, a concept central to this study, is influenced by subjective attitudes and the surrounding environment [25]. It refers to the behavioral propensity of tourists to assess their potential future actions from a subjective perspective. In this context, we redefine the concept of behavioral intention as the attitude and behavioral tendency of tourists after their tour [26]. The evaluation dimension of behavioral intention, as proposed by Chinese scholar Zou Bo, encompasses the probability judgment of tourists returning to the destination and recommending it to others [27].

The importance of behavioral intention in tourism research cannot be overstated. It effectively explains and reliably predicts the subsequent behaviors of tourists, making it a widely utilized concept in the field. This has sparked academic competition and active research, with scholars increasingly focusing on the factors that stimulate behavioral intention. They propose considering behavioral intention as an outcome variable and explore its relationship and influence mechanism with other variables [28, 29]. For instance, Fang Shumiao empirically discovered that higher perceived value correlates with a more prominent tendency for tourists to revisit or recommend the destination to others [30]. Gu Song et al. (2020) indicated that festival activities and place attachment are primary drivers for repeat visits. Liu Jingyan et al. (2015) revealed that tourist experience mediates the direct relationship between tourist mood and behavioral intention.

Despite these advancements, there remains a significant gap in understanding the relationship between landscape value perception in historical and cultural blocks and tourists’ behavioral intentions. To address this, our study introduces the concept of "place identity" from environmental psychology and human geography as a mediating variable. This approach allows for a more nuanced exploration of how landscape perception influences tourist behavior in these unique urban spaces.

Taking the historical block of Sifang Road, Zhongshan Road in Qingdao as our research domain, this study aims to:

1. Investigate the influence of perceived landscape value on tourists’ behavioral intentions.
2. Examine the mediating role of place identity in this relationship.
3. Provide practical implications for the sustainable development and conservation of historical and cultural blocks.

By revealing the correlation and structural relationships among landscape value perception, local identity, and tourists’ behavioral intentions in historical and cultural blocks, this research seeks to bridge the gap between theoretical understanding and practical application in urban heritage tourism. It offers valuable insights for urban planners, policymakers, and tourism managers in the sustainable management and development of these vital urban spaces. Moreover, this study contributes to the broader field of landscape perception and tourism behavior, providing a framework that can be adapted and applied to similar historical urban contexts worldwide.

## 2.Materials and methods

### 2.1 Study area

As a national historical and cultural city, Qingdao, Shandong Province, has a very special geographical location, characterized by "mountains, sea, island and city". In the "Qingdao Famous Historical and Cultural City Protection Plan (2011–2020)” the city is recognized as one of the most famous historical and cultural cities in China.

**Fig 1:**
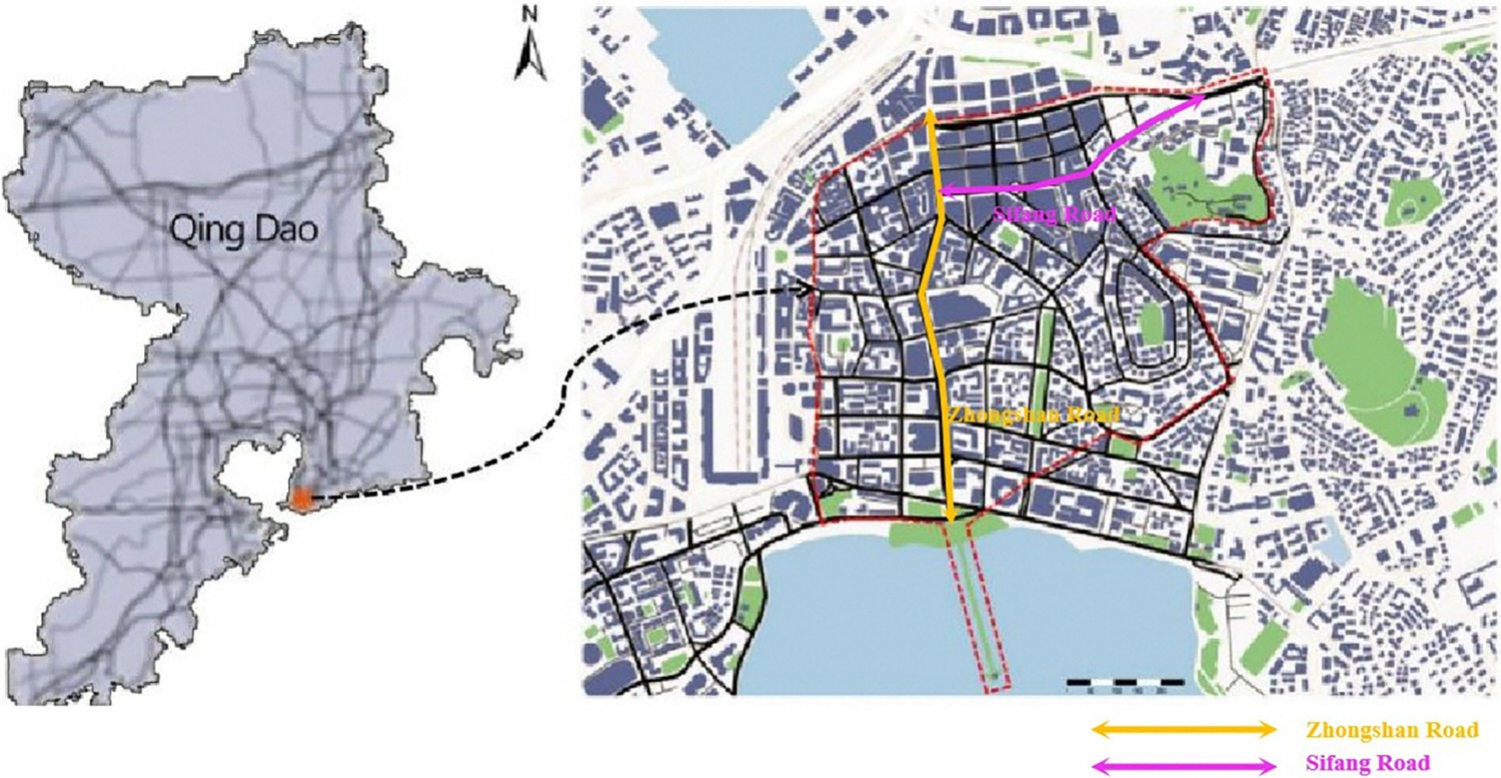
Zhongshan Road Sifang Road historical and cultural blocks.

The protection area of the Zhongshan Road and Sifang Road historical and cultural blocks totals 95 hectares (Fig 1).

**Fig 2:**
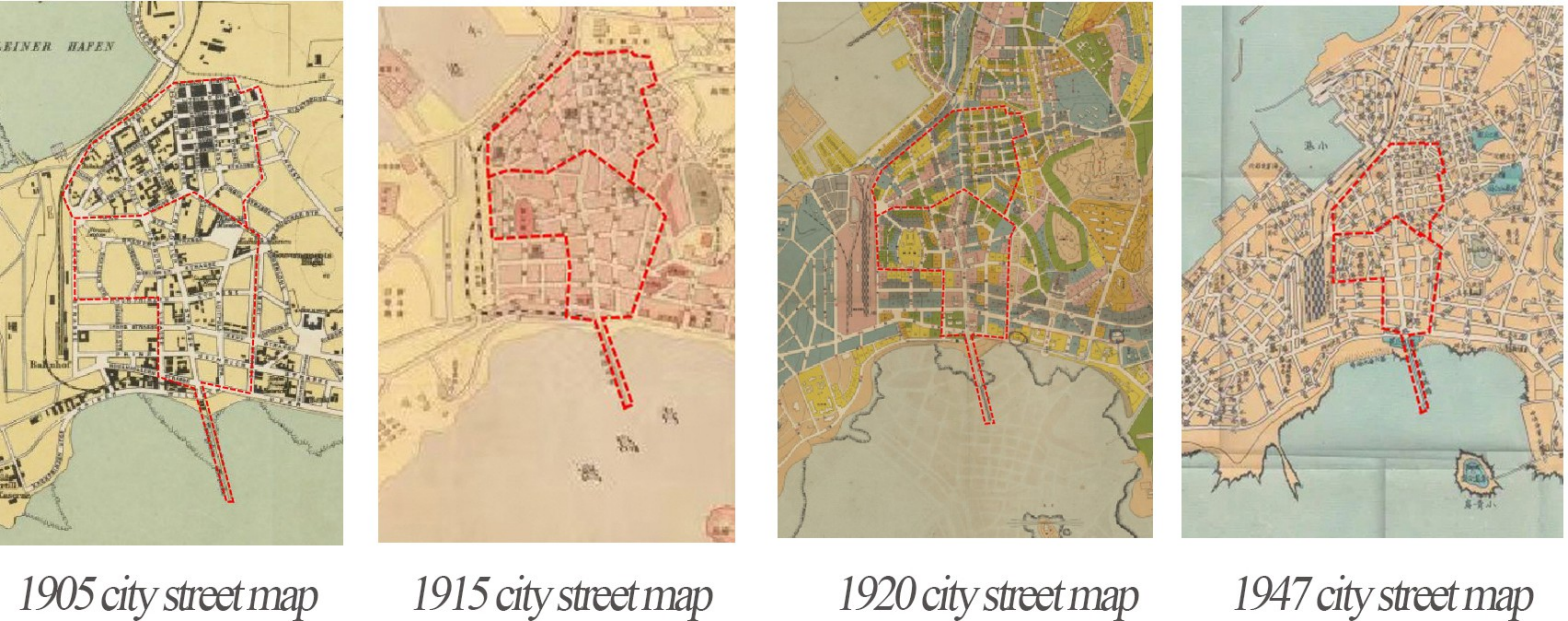
Qingdao Zhongshan Road Sifang historical and cultural block city street change map.

Qingdao Zhongshan Road and Sifang Road blocks were formed during the German occupation period and became the main axis of urban development (Fig 2).

The city of Qingdao, a Chinese-nationally recognized historical and cultural city, has designated 15 historic and cultural blocks, among which the Zhongshan Road and Sifang Road historic and cultural blocks embody the core values and unique historical features of the city. The Zhongshan Road historic and cultural block stands at the heart of Qingdao’s old town, marking the "starting point" of the city’s development and construction under the guidance of modern urban planning principles. This block is a traditional commercial hub that integrates shopping, dining, and entertainment, boasting a diverse array of businesses and forming a significant retail area within Qingdao’s historic urban area. Within this block, there are 29 cultural relics protection units, 15 historic buildings, and 79 traditional-style architectural structures. The Sifang Road historic and cultural block, on the other hand, is characterized by an abundance of "Liyuan" (traditional courtyard complexes), housing the largest concentration of such structures in Qingdao. The block boasts 7 cultural relics protection units, including Pichaiyuan, the former site of Sanjiang Guild Hall, and Chunhe building, along with 6 historic buildings, 215 traditional-style architectural structures, and 16 historic-style protected roads, including Zhongshan Road, all imbued with profound historical significance. The Zhongshan Road and Sifang Road historic and cultural districts showcase a rich tapestry of architectural styles, ranging from verandah-style, Gothic Revival, Classical Revival, to early modern designs, while also preserving a significant number of "Liyuan" buildings. Their unique street scales, architecture and courtyard texture, spatial characteristics and colors all perpetuate the characteristic history and culture of Qingdao, reflecting the prosperous commercial, life and cultural characteristics of Qingdao in the historical period.

**Fig 3:**
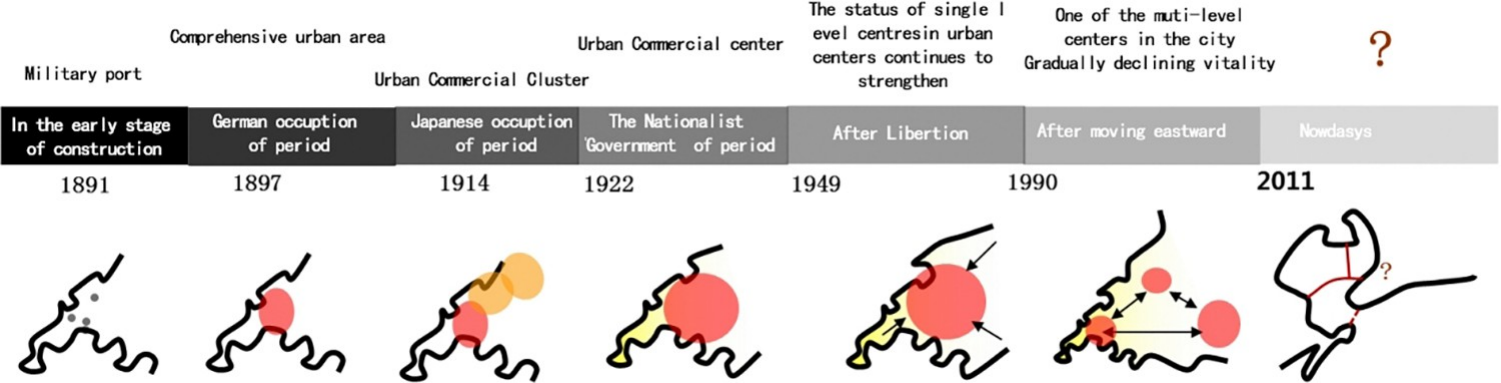
Functional evolution of Zhongshan Road Sifang Road.

The Qingdao Zhongshan Road and Sifang Road historical and cultural blocks are the centralized carrier and embodiment of Qingdao’s urban style, witnessing a hundred years of urban development history (Fig 3). Therefore, it is a typical case to explore the influence of landscape value perception on tourists’ behavioral intentions.

### 2.2 Data source and collection

#### 2.2.1 Online data

In order to ensure the comprehensiveness and authenticity of the data, and to avoid data limitations, the research data in this thesis were obtained by means of online network data. The "online review" data can maximize the collection of diversified tourists’ sample characteristics, regardless of the influence of national boundaries, geography, cultural level, values, etc., and has the independence to truly express the tourists’ perceptions [31]. The data were obtained from Ctrip.com and Qunar.com, which are the top ranked travel websites in China, and Dianping.com, the earliest independent third-party consumer review website in the world. After analyzing and finding that the selected websites have better platform data richness, credibility, and accessibility, we used Octopus Collector and python to collect and obtain the data of the reviews in the websites. In order to ensure the coherence and sample size of the network text data and reduce the impact of the new COVID-19 epidemic since 2020, the data comprising the network text were acquired from March 2019 to March 2023.

#### 2.2.2 Field research

Field research and interviews were used to obtain information on the age composition. The research area is located in the historical and cultural blocks of Sifang Road, Zhongshan Road, Shinan blocks, Qingdao. The interview period is from June 2023 to September 2023. During this period, three field visits will be conducted, each lasting 5–7 days. According to the structured observation plan, the research was organized in group. The research team consisted of 10 people and was divided into 5 groups with 2 people in each group. The members of the research team included planners, scholars, professional teachers and students. In order to ensure the diversity of views, sampling design is adopted to select interview subjects. First, 20 sampling indicators are assigned to two blocks of Zhongshan Road and Sifang Road respectively according to the size of historical and cultural blocks, and then purposive sampling is adopted to obtain samples. In the research field, 30 in-depth interviews were conducted in a semi-structured and open format, with each interview lasting 45 to 60 minutes. The questions involved information such as age, occupation and social income, as well as data such as perception content, perception measurement and behavior intention of the landscape value of historical and cultural blocks. Image collection by taking photos and information collection by writing records are adopted. The use of field research can obtain first-hand observations of the natural landscape and tourist behavior in the natural environment, and interviews can provide insight into the perceptions and experiences of tourists and local stakeholders. These research methods can complement quantitative data collection. The informed consent of respondents was obtained during the survey process and the information security of respondents was ensured. The survey process was supervised and approved by the ethics Review Committee.

#### 2.2.3 Questionnaire survey

In addition, in order to ensure the authenticity, diversity and breadth of the data, a network questionnaire was adopted to collect the scale data of landscape value perception, and the questionnaire was issued and collected from 6 June 2023 to 7 September 2023.All questionnaires were anonymised. The content of the questionnaire consists of three parts: the first part contains basic demographic characteristics, with basic questions consisting of gender, age, and geography; the second part is the focus of the research, including the landscape value perception scale, which contains four dimensions and twelve question items; and the third part is the behavioral intention scale. Each scale of the questionnaire was based on a 5-level Likert scale. A total of 500 questionnaires were distributed, all of which were returned, including 492 valid questionnaires, with a validity rate of 98.4%. The questionnaires were completed with high quality.

### 2.3 Data analysis

The content analysis method is employed for semantic segmentation and word frequency purification of the text to prepare for coding in grounded theory research. The network connection and affective attitude among semantics are analyzed to identify the perceptual linkage between landscape value perception and behavioral intention. Secondly, with the assistance of grounded theory and the structural equation model, the intricate relationship between landscape value perception and behavior intention of historical cultural blocks is explored. Grounded theory can leverage original text data, conduct open coding, and extract core concepts and categories therefrom, establish a theoretical framework closely related to the research object, and investigate the dimensions of landscape value perception of historical and cultural blocks from a qualitative perspective. The structural equation model can simultaneously reveal the relationship between multiple latent variables and observed variables. Through path analysis and model fitting, it scientifically validates the hypotheses and relationships of grounded theory and utilizes quantitative methods to ensure the accuracy and reliability of research outcomes. In conclusion, the application of the aforementioned research methods can disclose the complex relationship between the perception of landscape value of historical cultural blocks and tourists’ behavioral intention, ranging from perceptual research to rational research and from qualitative research to quantitative research, laying a solid theoretical foundation for drawing precise and scientific conclusions.

#### 2.3.1 Content analysis method

With the help of Rost CM6.0, the text data, vocabulary cut and word frequency statistics [32–34] were analyzed on the preprocessed text. The core elements of the historical and cultural block were extracted and a semantic network was constructed. Then, the relationship between the core elements of landscape perception was analyzed. With the help of Rost EA, tourists’ emotional tendency and perceived attitude and the correlation between tourists’ landscape value perception and emotional tendency were analyzed.

Preliminary data cleaning of the collected text was completed with deletion of invalid comments, such as "great, good" and other comments that only expressed the emotion of the text without research significance; the use of RostCM6.0 on the effective text for vocabulary cutting and word frequency statistics, eliminating virtual words that were meaningless for the study, such as "already", "located", "look"e.g.; and organizing the text in the occurrence of high-frequency words (Table 1).

**Table 1:** List of high-frequency words.

Ranking	High-Frequency vocabulary	Frequency	Ranking	High-Frequency vocabulary	Frequency
1	Qingdao, subprovincial city in Shandong	7558	81	aesthetics	351
2	gull	6509	82	piazza	307
3	a loading trestle for goods or passengers	4759	83	sino	302
4	flat-roofed building	2513	84	beautiful	299
5	constructions	2190	85	code	246
6	(euphemism) go to the toilet	2121	86	prestigious	230
7	Catholic Church	1582	87	timing	217
8	transportation	1554	88	tourists	202
9	watch the sea	1533	89	gourmet food	187
10	landscaping	1259	90	drawing	177
11	neighboring	1199	91	navies	175
12	iconic	1078	92	liberalization	170
13	friends	1072	93	Island	160
14	sea breeze	1065	94	flavor	155
15	number one	1053	95	University Avenue	155
16	pull up (stop one’s vehicle)	1035	96	an experience	151
17	sandy shore	1018	97	exterior condition	150
18	in winter	1017	98	good-looking	148
19	dissatisfied	1016	99	interestingly	142
20	metro	1012	100	be gluttonous	140
21	favorable criticism	1012	101	typical case	127
22	be spared worry	1000	102	scenery	126
23	ticket (for theater, cinema etc)	988	103	old location	125
24	place of interest (tourism)	936	104	Romanesque	125
25	nice	914	105	picturesque	109
26	German	842	106	well-known	110
27	free (of charge)	740	107	ambitious	111
28	seafront	734	108	designer	112
29	park (for public recreation)	713	109	station	113
30	suitability	703	110	roam around	114
31	quality-price ratio	682	111	European	115
32	travelers	663	112	solemnity	116
33	cays	609	113	picturesque	109
34	scenic area	601	114	well-known	110
35	dolphin	597	115	ambitious	111
36	placement	586	116	designer	112
37	experience for oneself	582	117	station	113
38	lovers	570	118	roam around	107
39	climatic	568	119	European	103
40	moods	558	120	solemnity	102
41	service	553	121	Welcome Hotel	98
42	color	549	122	ancient castle	98
43	roomy	546	123	grill	97
44	peak season	544	124	Goddard (name)	95
45	green sea	537	125	artists	95
46	facilitation	528	126	gothic	95
47	levees	525	127	hundred years	94
48	mass transit	522	128	sacrosanct	91
49	go on foot	521	129	German style	90
50	season	520	130	admiration	89
51	cost-effective	519	131	explain	89
52	engage in physical exercise	513	132	age	88
53	snacks	511	133	cheap	86
54	distinctiveness	510	134	go sight-seeing	85
55	hairstyle	510	135	romantic	84
56	histories	510	136	Rickshaw Boy	82
57	museums	509	137	iconic	82
58	Lu Xun (1881–1936), one of the earliest and best-known modern Chinese writers	508	138	magnificent sight	79
59	Zhongshan Road	155	139	quietly	77
60	warmer	155	140	aesthetics	77
61	exposure	151	141	ornamental	77
62	former residence	150	142	shopping streets	75
63	stir up (emotions)	148	143	literature and art	75
64	traffic jam	142	144	enjoyable	74
65	lit. rubbing shoulders and following in each other’s footsteps	140	145	historical center	74
66	delightful	139	146	shrimp	74
67	a tide of people	133	147	shopping streets	75
68	attentive	127	148	take a stroll	73
69	gush	501	149	Euclidean	72
70	billow	501	150	charging	72
71	recommend	500	151	beacon	70
72	metro entrance	500	152	south city block	70
73	seasonality	500	153	seafront	70
74	bustling with noise and excitement	476	154	small store	69
75	wedding photos	470	155	blue sky	69
76	take a picture	435	156	magnanimity	68
77	lit. split wood courtyard	428	157	elegance	68
78	look around	380	158	water	67
79	matrix	379	159	Catholicism	67
80	seafood	435	160	metrology	65

The network text is divided into words and cut, and the semantic network is linked by Rost CM6.0. The semantic network can reflect the interconnection of the landscape perception content in the historical and cultural blocks; the analysis of emotional tendency mainly analyzes the emotional nouns and adjectives used in the network text, the emotional tendency reacts to the tourists’ behavioral intentions in the historical and cultural blocks [35]. The emotional tendency can be divided into positive emotional attitudes, neutral attitudes, and negative emotional attitude. Positive emotional attitude will inevitably bring strong behavioral intentions, and negative emotional attitude will affect the performance of behavioral intention. It can also identify the landscape value perception content that triggers the positive or negative emotional tendency.

#### 2.3.2 Grounded theory

Grounded theory was proposed by the American sociologists Glaser and Strauss in 1967, based on the comparison, categorization and connection of primary sources, seeking to reflect the core concepts of social phenomena [36]; then, constructing the relevant social theories bottom-up through the connection between concepts. In this paper, we applied the procedural grounded theory to code the review texts of historical neighborhoods step by step, including open coding, spindle coding and selective coding [37].

Internet comment text is usually able to respond to the real value of the interaction between the tourists’ perception and the destination [38]; based on analysis of high-frequency vocabulary in the Internet comment text, it directly responds to the convergence of the tourists’ perception of the specific landscape value of historical and cultural blocks. With the help of the theoretical basis of the previous paper, we used the grounded theory of coding and parsing of the Internet comment data to identify the value of the landscape of the historical and cultural blocks. Therefore, based on the previous theoretical research, we used the grounded theory to code and analyze the online review data to identify the landscape value of historical and cultural blocks, which is divided into three major processes: open coding, spindle coding and selective coding.

1. Open coding
  Open coding is the process of conceptualizing and categorizing raw materials, and its principle is to be free from the influence of established facts and personal concepts. The word-by-word parsing was used to repeatedly compare and read the collected primary materials, then integrate and refine them after extracting them from the high-frequency text to form conceptual categories, and finally generalize them to get nine main categories. As shown in Table 2.
2. Main-axis coding
  In order to explore the intrinsic connections between the open codes, the conceptual categories need to be further refined to summarize the more refined main categories, i.e., the spindle codes. The nine conceptual categories were sorted and combined again to obtain the main categories with internal logical relationships.
  The nine conceptual categories are sorted and combined again, and the main categories with intrinsic logical relationships are organized. Through summarization, four core categories were finally obtained, namely, ecological landscape value, historical and cultural value, skill education value, and recreational value. The content of the main axis code is shown in Table 2.
3. Selective coding
  Selective coding is a method to find the intrinsic path relationships between main categories from logical relationships after completing the coding of main axes. Theory saturation is by analyzing new data outside of the sample data, and it is also not possible to obtain new concepts, categories, and further development of a category. Based the basic idea of grounded theory, this study processed the sample data reserved for 1/3 of the network evaluation with the three-level coding of grounded theory to test the theoretical saturation of the conceptual model of the core categories of the perceived value of the landscape of the historical and cultural blocks, and categorized the main categories and high-frequency vocabulary derived from the grounded theory in the previous section as a reference for the current analysis. Through repeated generalization and analysis of new materials, comparing the second coding results with the first coding results, no new conceptual categories appeared, and it was not possible to add to the existing categories, and the nine main categories mentioned above almost covered the factors affecting tourists’ perception of landscape value in the historical and cultural blocks, which can be judged that the "conceptual model of landscape value perception in historical and cultural blocks" is nearly saturated. Thus, it can be judged that the theory of "conceptualization model of landscape value perception of historical and cultural blocks" is close to saturation and can effectively reflect the type of landscape perception.

**Table 2:** Encoding of information.

Core scope	Main category	Original text (high-frequency vocabulary)
Perceived ecological landscape value	Seaside View	pier, seaside, beachfront, seagulls, sea breeze, sea water, pier, reef, looking at the sea, seascape, waves, yacht, coast, beach, lighthouse, shells, high tide, sunrise, waves, coastline, catching the sea. . .. . .
	Park View	pleasant, landscaping, garden style, plants, flowers, parks, sculpture. . .. . .
	natural landscape	Wet, cool, pleasant, four distinct seasons, warm winters, cool summers, fresh air. . .. . .
Perception of historical and cultural values	remains	Lyceum, architecture, centennial, German prison, Romanesque, octagonal, flowery windows, official residence, Italianate, Gothic, architectural layout, old site, guildhall, relics, governor’s residence. . .. . .
	humanities and customs	Xiangzi Camel, Lu Xun, Wen Yiduo, sugar painting, paper cutting, social theater. . .. . .
Perceived value of skill education	Skill development	Design skills, sketching, mapping
	Educational inspiration	Architecture, history, aesthetics
Perceived value of leisure and recreation	entertainment	Taking pictures, hitting the beach, swimming, watching the sea, walking, shopping, visiting, listening to the sea, blowing in the wind. . .. . .
	infrastructure	Subway, accommodation, commerce, specialty products, snacks, tickets, souvenirs

In this study, the nine main categories are generalized and adjusted to form four core categories that can comprehensively express the landscape perception of historical and cultural blocks.

The sample data of the price were processed by the three-level coding of the grounded theory, and the theoretical saturation test was carried out on the conceptual model of the core categories of the landscape value perception of the historical and cultural blocks. The main categories and high-frequency vocabulary derived from the grounded theory in the previous section were used as references to be categorized in the present analysis. Through repeated generalization and analysis of the new material, comparing the second coding results with the first coding results, no new conceptual categories appeared, and it was impossible to add to the existing categories. The nine main categories mentioned above almost covered the factors affecting tourists’ perception of landscape value in the historical and cultural blocks, through which it can be judged that the theory of the "conceptualization model of landscape value perception of historical and cultural blocks" is close to saturation and can effectively reflect the type of landscape perception.

#### 2.3.3 Structural equation modeling

**Fig 4:**
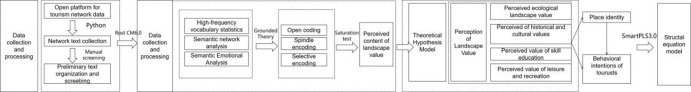
Research design.

Structural equation modeling software AMOS 24.0 and statistical software SPSS 25.0 were used for the quantitative analysis of data. The model is not only able to express the causal relationship graphically, but also able to detect multiple variables and the internal structure of variables and their correlations [39]. Using the model to evaluate the dimensions of perceived landscape value of historical and cultural blocks, it is able to portray the correlation between potential variables in a more in-depth manner.(Fig 4)

## 3.Results and discussion

### 3.1 Sample characteristics

Analysis of the collected samples: Men accounted for 48.1%, women accounted for 51.8% of the sample; the age of the respondents was concentrated in the 26–36 years old age group and accounted for 36.18%; the specific distribution (Table 3) shows that the distribution of the respondents contains 17 provinces and cities in China; the data are mainly concentrated in Shandong Province which accounted for 60.73%. Table 4 shows that the sample of the overall data collection is more comprehensive, includes the majority of sample characteristics, and has research value.

**Table 3:** Age distribution of respondents.

Age distribution	proportion
18–25	26.02%
26–36	36.18%
37–50	23.78%
50 or more	14.02%

**Table 4:** Regional distribution of interviewees.

Regional distribution of interviewees	Number of interviewees
Shandong Province	387
Jiangsu Province	24
Ahejiang Province	14
Anhui Province	9
Hubei Province	6
Guangdong Province	6
Fujian Province	6
Heilongjiang Province	5
Liaoning Province	4
Heibei Province	3
Liangxi Province	2
Yunnan Province	2
Jilin Province	2
Hunan Province	1
Guizhou Province	1
Xizang Province	1

The 110 interview samples obtained from the resident population of the historical and cultural blocks included 47.01% of people over 50 years of age and 28.73% of people between 30 and 40 years of age; the composition of the occupational structure included 38.60% of people engaged in other professions, 32.46% of retired residents, 20.18% of residents engaged in individual businesses, and 8.77% of office workers.

### 3.2 Semantic network analysis of landscape value perception

**Fig 5:**
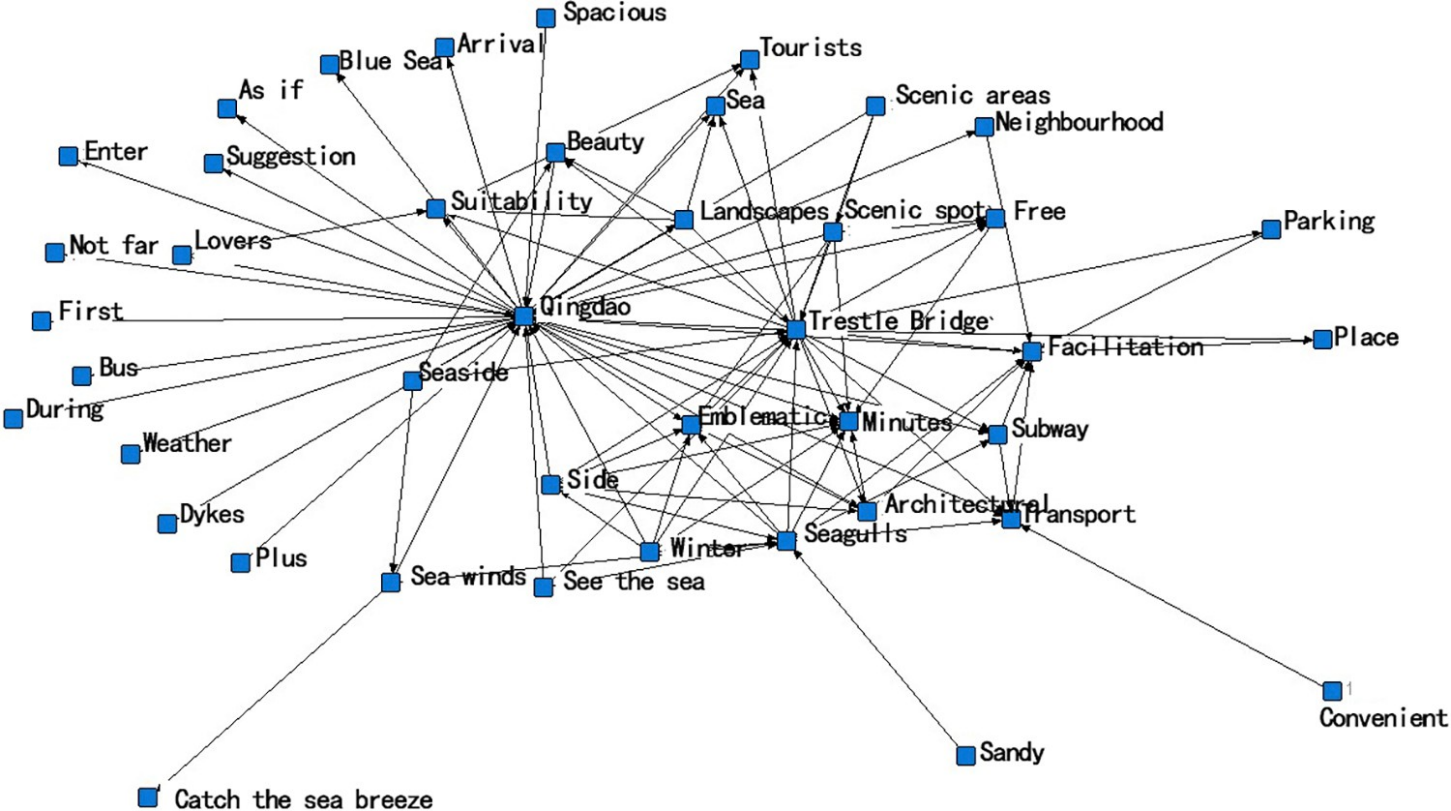
Semantic network analysis.

The semantic network formation process summarizes the fragmented high-frequency words and their interconnections, as shown in (Fig 5). In the network structure, it contains the content of landscape perception of historical and cultural blocks, starting from the two core points of Qingdao region and Trestle Bridge, and unfolding the related activities and perceptual content of landscape perception from the center. In addition to the core elements, there are also a large number of edge elements, whose perception frequency is low or do not exist in the semantic network, indicating that the perception intensity of tourists is low [40]. With the help of a semantic network analysis diagram, it can help to summarize and conclude the content system of landscape value perception of historical and cultural blocks, and reflect the strong and weak relationships and interconnection of the content of tourists’ perception of landscape value [41].

### 3.3 Emotional characteristics of historical and cultural blocks

The emotional tendency and perception attitude of tourists’ landscape perception of historical and cultural blocks are shown in Table 5. We further analyzed the landscape perceptions contained in the network texts of positive and negative emotions, organized and summarized the negative and positive emotional texts, and carried out preliminary identification and classification of some of the landscape perceptions (Table 6). ROST EA 1.9.0.4 was used to analyze the emotional tendency of the network text, and the analysis shows that most of the semantic identification of negative attitudes comes from leisure and entertainment value perception. Most of the positive emotional attitudes come from historical and cultural value perception and skill and educational value perception.

**Table 5:** Emotional tendencies of web texts.

emotional disposition	positive emotions	neutral mood	negativity	Total number of statements
Aggregate situation	66.98%	4.30%	28.72%	13795

**Table 6:** Sentiment analysis of web texts.

emotional attitude	sentimental value	Likert scale	Text content	Types of perceived landscape value
Negativity Emotions	-32.0	-3	It’s not worth a visit, it’s not even an attraction, just a few walls and graffiti. I don’t know which netizen took a few random photos to attract them to go there. Parking nearby is difficult and expensive.	Perceived value of leisure and recreation
	-12.8	-1	Wouldn’t recommend driving, if you do park in the university road parking lot. 10 bucks an hour with no limit. . .I parked for 40 bucks the other day. . .heartbreaking!	Perceived value of leisure and recreation
	-13	-1	A must-visit place for literary youngsters, and as a reminder, don’t go with a suitcase. Because the road is in inconvenient to carry.	Perceived value of leisure and recreation
	-7.20	-1	I went here specifically when I went to Qingdao to play, but it’s not interesting at all, not recommended!	Perceived value of leisure and recreation
	-0.80	0	Not far from the scenic area of an old snack street, not too long, but more businesses, fewer tourists, formerly famous now fallen, strolling around, did not want, although the price is not expensive, seafood-based fear.	Perceived value of leisure and recreation
	-6.68	-1	Here is not as good as expected, a bit like the section of Xi’an Hui Min Street, inside the lively and noisy shouting and selling, went to the Jiangning Hall, pick your own seafood, according to taste processing, there are performances but at 8:00 p.m. on the end!	Perceived value of leisure and recreation
	-8.0	1	Commercial atmosphere is too strong, did not eat inside the various grades of restaurants do not delusion.	Perceived value of leisure and recreation
	-6.4	-1	Spent over a hundred dollars on a taxi to go there, but it turned out to be a small alley, a dozen small stores, the food looks bad, not interesting at all, next time will definitely not go. . .. . .	Perceived value of leisure and recreation
	-12.72	-1	A very beautiful church, the church before the small square a lot of wedding photos, this is also a must in Qingdao, Qingdao’s old city landmarks, there is just a little bit of feeling not so good, the entrance to the square in front of a lot of cars parked in a mess, in other cities, the square in front of the tourist attractions should not be allowed to park, probably because of the old city parking place is too little.	Perceived value of leisure and recreation
	-18.78	2	The exterior of the building is still quite nice, typical of the Gothic style, and there is a charge if you go inside, ten dollars, which is still a particularly low price, because many churches are actually free to enter.	Perceived value of leisure and recreation
Positive Emotions	43.7	3	It is worth a visit, driving can park in the parking lot near the Huangxian Branch Road, to the north to see after the Governor’s House, you can turn around to the south, the old residence, the red wall, all the way to the scenery is good, a lot of old German buildings are still in use, the old residence of the old residence exit has a bookstore is good to buy a book can be stamped, to the child is quite meaningful.	Perception of historical and cultural values
	28.4	3	A very refreshing road, it is also from the Internet and then came to the name, probably in July for the sake of the university road is almost no one, there is no car, basically on both sides of the decoration style is very fresh or very personalized cafe and some small stores selling accessories, find three or five friends, or one person, find a cafe, take pictures, chat, will be very comfortable!	Perceived value of leisure and recreation
	19	2	The purely German architecture, with courtyards, staircases, rooms and halls, is worth a closer look.	Perception of historical and cultural values
	16	2	Strolling through the old streets, the city’s smoky atmosphere and anime-like richness of colors are intertwined in a romantic and vivid way.	Perceived value of leisure and recreation
	16.1	2	This historic old house is over a hundred years old, and even now, it’s distinctive and not at all dated.	Perception of historical and cultural values
	68.1	3	Governor building architecture is very shocking, like a beautiful work of art, reflecting the East and West culture in this collision and fruitful, and other people pay attention to his internal furnishings, I appreciate more is the exterior of the building to give people a kind of noble, the atmosphere of extraordinary visual impact, it is worth a look.	Perception of historical and cultural values
	7.5	1	It’s very interesting, basically tells the story of the building from its birth to the present day, and introduces other knowledge like cutlery and such as well, which is interesting. The descriptions of dragon veins and such were also interesting.	Perception of historical and cultural values
	13	1	It’s worth a visit to Qingdao, where the German-style buildings still shine after a century of weathering.	Perception of historical and cultural values
	3.3	2	There are historical and cultural places to visit and learn with your kids!	Perceived value of skill education
	4.2	0	Very European town feeling, around is also a special literary place, pink spires like fairy tale castles, very many couples in the photo shoot wedding, around the cafe ah store ah are very feeling, very literary Oh!	Perceived value of leisure and recreation

### 3.4 Relationship between perceived landscape value and visitor behavioral intentions

**Fig 6:**
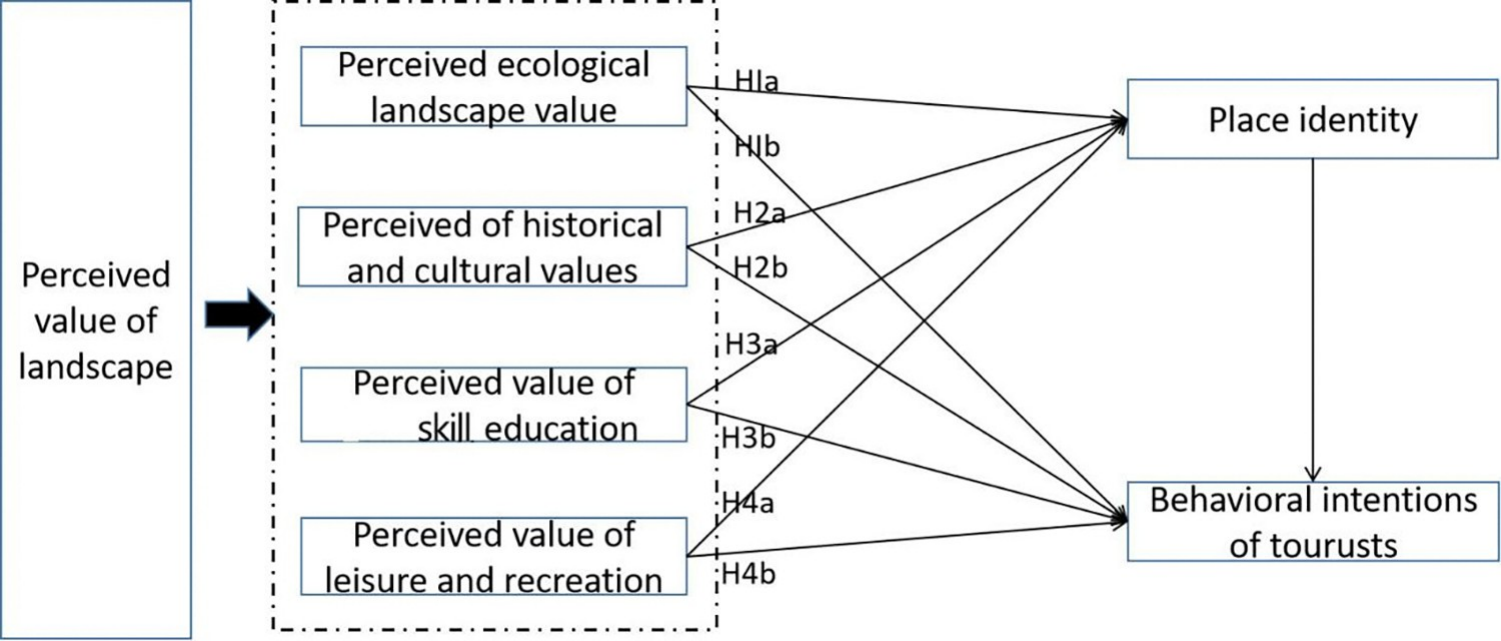
Theoretical hypothetical model.

Landscape value perception is multidimensional change, and the division of its dimensions is not the same [42–46]. This paper adopts the research method of grounded theory to classify four dimensions of landscape value perception for the characteristics of historical and cultural blocks: ecological landscape value perception, historical and cultural value perception, skill and education value perception, and leisure and entertainment value perception. historical and cultural blocks, as the bearing space of local characteristics of history and culture, are a unique window into the history of the city [47]; there is a correlation between the landscape value perception of the neighborhoods and the behavioral intentions of tourists, and the research on historical and cultural blocks of the behavioral intentions of tourists is relatively scarce. Therefore, it is necessary to explore the relationship between landscape value perception and tourists’ behavioral intentions based on the four dimensions of landscape value perception in historical and cultural blocks classified in the previous study. Place identity is chosen as a mediating variable in the research process, and the formation of place identity is the result of the interaction and influence between different individuals’ internal and external environments, which is able to map the subject’s emotional connection and significance of the featured place. Based on this, the following hypotheses are proposed (Fig 6):

- Perceived Landscape Value and Visitor Behavioral Intentions:
  H1 The perceived ecological landscape value has a significant positive effect on tourists’ behavioral intentions.
  H2 The perceived historical and cultural value has a significant positive effect on tourists’ behavioral intentions.
  H3 The perceived value of skill and education has a significant positive effect on tourists’ behavioral intentions.
  H4 The perceived value of leisure and recreation has a significant positive effect on tourists’ behavioral intentions.
- Local Identity and Perceived Landscape Value:
  H5 The perceived ecological landscape value has a significant positive effect on place identity.
  H6 The perceived historical and cultural values have a significant positive effect on place identity.
  H7 The perceived value of skill and education has a significant positive effect on place identity.
  H8 The perceived value of leisure and recreation has a significant positive effect on place identity.
- Place Identity and Tourist Behavioral Intentions:
  H9 Place identity has a significant positive effect on tourists’ behavioral intentions.
  H10 Local identity mediates the effect of perceived ecological landscape value and tourists’ behavioral intentions.
  H11 The mediating role of local identity in the influence of perceived historical and cultural values and tourists’ behavioral intentions.
  H12 The mediating role of place identity in the influence of perceived value of skill and education and tourists’ behavioral intentions.
  H13 The mediating role of place identity in the influence of perceived recreational value and tourists’ behavioral intentions.

#### 3.4.1 Data analysis and research hypotheses

Reliability analysis of the data using the statistical software spss25.0 showed that the coefficient of Cronbach’s alpha for each latent variable was in the range of 0.831–0.858, the KMO value of the sample data was 0.987, and the Bartlett’s Sphericity Test examined the significant difference value of 0.000, so the question items have a high level of confidence.

The validity test is mainly conducted in three aspects: content validity, distinction validity and convergence validity. Content validity is based on the design of the questions based on the grounded theory and references, and at the same time, it can be recognized that the content validity is high; according to the standardized factor loadings corresponding to each variable, the results are mainly concentrated in the range of 0.850–0.893, and the average variance extracted (AVE) value of each potential variable is in the range of 0.749–0.779, all higher than 0.7, greater than the critical value of 0.5. The combined reliability (CR) is in the range of 0.899–0.914, all greater than 0.7, with a high degree of convergent validity, as shown in Tables 7 and 8. The coefficients of explanation of each observed variable for the latent variables, as shown in Table 9, are all higher than 0.8, which is greater than its critical value of 0.5. The corresponding significance level P-values are 0.000 (Table 10), less than the critical value of 0.005; at the same time, according to the analysis of differential validity, it can be seen that the value of the diagonal is greater than the value of the flush below, so the question item has a high differential validity (Table 11).

**Table 7:** Reliability and validity analysis.

	Cronbach’s Alpha	rho_A	Combined Reliability (CR)	Average Extraction Variance (AVE)
Perceived value of leisure and recreation	0.837	0.841	0.902	0.754
Perception of historical and cultural values	0.831	0.832	0.899	0.748
local identity	0.848	0.851	0.908	0.767
Perceived value of skill education	0.852	0.857	0.91	0.772
Perceived ecological landscape value	0.849	0.853	0.908	0.768
Willingness to act	0.858	0.858	0.914	0.779

**Table 8:** External model load matrix1.

	Perceived value of leisure and recreation	Perception of historical and cultural values	local identity	Perceived value of skill education	Perceived ecological landscape value	behavioral intention
A1					0.85	
A2					0.892	
A3					0.886	
B1		0.852				
B2		0.862				
B3		0.88				
C1				0.852		
C2				0.89		
C3				0.893		
D1	0.852					
D2	0.861					
D3	0.892					
X1			0.857			
X2			0.888			
X3			0.881			
Y1						0.868
Y2						0.89
Y3						0.89

**Table 9:** External model load matrix 1.

	Perceived value of leisure and recreation	Perception of historical and cultural values	local identity	Perceived value of skill education	Perceived ecological landscape value	behavioral intention
A1					0.85	
A2					0.892	
A3					0.886	
B1		0.852				
B2		0.862				
B3		0.88				
C1				0.852		
C2				0.89		
C3				0.893		
D1	0.852					
D2	0.861					
D3	0.892					
X1			0.857			
X2			0.888			
X3			0.881			
Y1						0.868
Y2						0.89
Y3						0.89

**Table 10:** External model load matrix 2.

	Initial sample (O)	Sample mean (M)	Standard deviation (STDEV)	T-statistic (|O/STDEV|)	P-value
A1 < Perceived ecological landscape value	0.85	0.85	0.013	67.743	0
A2 <- Perceived ecological landscape value	0.892	0.892	0.009	98.219	0
A3 <- Perceived ecological landscape value	0.886	0.886	0.011	83.01	0
B1 < Perception of historical and cultural values	0.852	0.852	0.014	60.457	0
B2 <Perception of historical and cultural values	0. 862	0.861	0.012	74.166	0
B3 <Perception of historical and cultural values	0.88	0.88	0.009	94.479	0
C1 <Perceived value of skill education	0.852	0.852	0.013	64.167	0
C2 <Perceived value of skill education	0.89	0.89	0.01	91.308	0
C3 <Perceived value of skill education	0.893	0.894	0.008	109.419	0
D1 < Perceived value of recreation	0.852	0.851	0.013	64.039	0
D2 <Perceived value of recreation	0. 861	0.861	0.012	69.437	0
D3 <Perceived value of recreation	0.892	0.892	0.009	103.419	0
X1 <Local identity	0.857	0.856	0.013	66.495	0
X2 <Local identity	0.888	0.888	0.009	103.713	0
X3 <Local identity	0.881	0.881	0.01	87.034	0
Y1 <- Behavioral Intent	0.868	0.868	0.01	85.172	0
Y2 <- Behavioral Intent	0.89	0.89	0.009	100. 999	0
Y3 <- Behavioral Intent	0.89	0.89	0.008	111.386	0

**Table 11:** Distinctive validity analysis.

	Perceived value of leisure and recreation	Perception of historical and cultural values	local identity	Perceived value of skill education	Perceived ecological landscape value	behavioral intention
Perceived value of leisure and recreation	0.868					
Perception of historical and cultural values	0.828	0.865				
local identity	0.829	0.843	0.876			
Perceived value of skill education	0.833	0.847	0.843	0.879		
Perceived ecological landscape value	0.825	0.856	0.837	0.841	0.876	
behavioral intention	0.845	0.861	0.858	0.867	0.85	0.883

#### 3.4.2 Structural model fitness testing

The overall fitness index should be tested when the model is evaluated. As can be seen from Table 12, the coefficient of determination R^2^ of place identity is 80%, and R^2^of behavioral intention is 84.3%, the R^2^ value indicates the model’s ability to explain the variation of the endogenous latent variables, and the higher the R^2^ value indicates that the model explains almost all of the variation, where the R^2^ values are all greater than 0.67, and the perceived landscape value is a stronger explanation of the place identity and the behavioral intention. The SRMR value is used to assess the observation and expectation matrix The average size of the differences, SRMR is less than 0.08, the normative fit indicator NFI value is between 0–1, which is equal to about 0.9, the model has a good fit.

**Table 12:** Distinctive validity analysis.

	R^2^	Adjusted R2	model fitness
local identity	0.8	0.798	SRMR: 0.048 NFI: 0.87 rms Theta: 0. 178
behavioral intention	0.843	0.841	

### 3.4.3 Hypothesis testing

**Fig 7:**
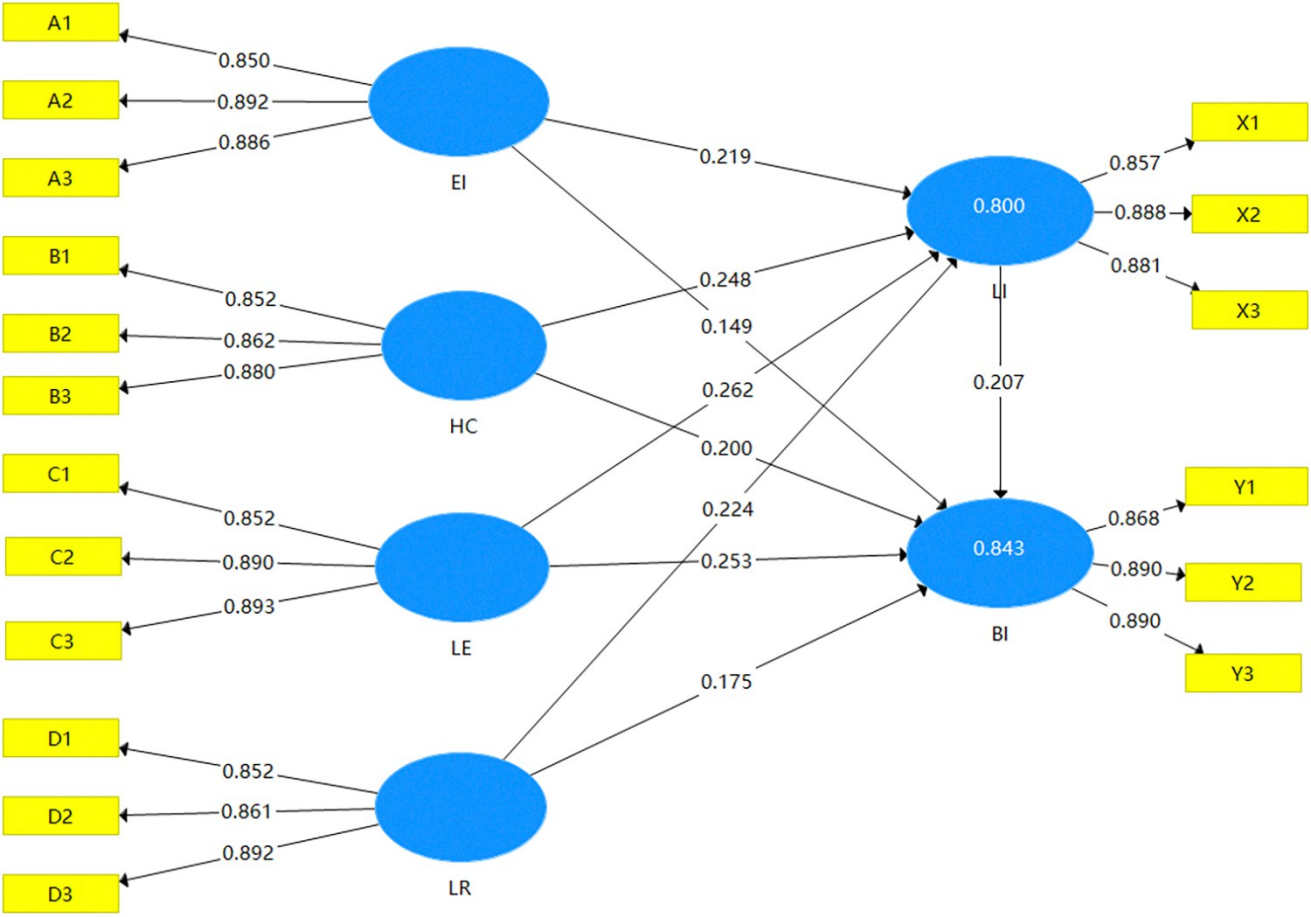
Structural equation model.

The structural equation modeling relationship paths generated by SmartPLS (Fig 7) explain the degree of influence and structural relationships between the values of the latent variables in the theoretical hypotheses model constructed in the previous section.

The PLS algorithm and consistency algorithm were performed on the model paths for modeling, the individual path coefficients, and the significance levels were also obtained, as shown in Table 13.

**Table 13:** Distinctive validity analysis.

pathway relationship	Initial sample (O)	Sample mean (M)	Standard deviation (STDEV)	T-statistic (|O/STDEV|)	P-value
Perceived value of recreation > Local identity	0.224	0.225	0.046	4.845	0. 000
Leisure Value Perception > Behavioral Intentions	0.175	0.174	0.041	4.264	0.000
Perception of historical and cultural values> Local identity	0.248	0.248	0.045	5.522	0.000
Perception of Historical and Cultural Values> Behavioral Intentions	0.200	0.202	0.043	4.682	0.000
Local Identity> Behavioral Intentions	0.207	0.204	0.044	4.654	0.000
Perceived value of skill education> Local identity	0.262	0.262	0.049	5.301	0.000
Perceived Value of skill education> Behavioral Intentions	0.253	0.255	0.041	6.102	0.000
Ecological landscape value> Local identity	0.219	0.218	0.046	4.780	0.000
Ecological Landscape Value> Behavioral Intentions	0.149	0.148	0.040	3.701	0.000

From the results of PLS path analysis, it can be seen that the path coefficients of leisure and recreation value perception on local identity and behavioral intentions are 0.224, 0.175, with a T-value > 2.58, significance level P-value < 0.01; therefore, the hypotheses of the previous H1a and H1b are valid. The path coefficients of history and culture value perception on local identity and behavioral intention are 0.248, 0.200 and the T-value is >2.58, significance level P-value <0.01; therefore, the validation hypothesis of H2a and H2b is established. The path coefficients of the value perception of education and learning on the local identity and behavioral intention are 0.262 and 0.253, respectively, and the T-value is >2.58, and significance level P-value <0.01; therefore, the validation hypotheses of H3a and H3b are established. The path coefficients of intention are 0.219, 0.149, the T-value is >2.58, significance level P-value <0.01, so the validation hypotheses of H4a and H4b are established.

Local identity as a mediating variable was validated in SmartPLS using the VAF (Variance account for) value for testing the mediating effect, calculated using the following formula:

$$VAF=\frac{a*b}{a*b+c}*100\%$$

The VAF values of education and learning value perception and ecological landscape value perception are less than 20%, so there is no mediating effect of local identity as a mediating variable. The VAF values of recreation and entertainment value perception and historical and cultural value perception are greater than 20% and less than 80%, which can be judged as a partial mediating effect of local identity (Table 14). Therefore, it is assumed that hypotheses H10 and H12 in the model are not valid, and H11 and H13 are not fully valid.

**Table 14:** Mediated effects test.

	a	b	c	VAF
Perceived value of skill education > Local identity > Behavioral intention	0.262	0.207	0.253	17.65%
Ecological landscape value perception > Local identity > Behavioral intention	0.219	0.207	0.248	15.45%
Perceived value of recreation > Local identity > Behavioral intention	0.253	0.207	0.175	23.03%
Perception of historical and cultural values > Local identity > Behavioral intention	0.248	0.207	0.200	20.42%

### 3.5 Discussion of research finding

1. From the basic questionnaire part, it can be seen that 60.45% of the people participating in the research questionnaire are people from Shandong Province other than Qingdao, and people from outside Shandong Province only account for 21.91%. The tourists in Qingdao’s historical and cultural blocks are mostly concentrated within Shandong Province, and mainly focus on Shandong provincial tours, with fewer tourists from outside the province. The 100 households in the historic and cultural blocks have a high degree of aging, unemployment problems, a large proportion of low-income people, and the residents have a low perception of the landscape value of the historic and cultural blocks. The composition of human settlements and the living conditions of the historic and cultural blocks are a microcosmic manifestation of the effectiveness of the block’s regeneration and development, which has been examined from the perspective of the planner’s professionalism, the management of governmental departments, and the tourism and recreation perspective. In previous studies, the renewal and reform development of historical and cultural blocks was carried out from the professional perspective of planners, the management perspective of government departments, and the recreational perspective of tourists. Insufficient attention has been paid to the living demands and value perception of the residents living in historical and cultural blocks. The main reason is that historical and cultural blocks are viewed as being mainly for the function of tourism. In the development of neighborhoods, the renewal and vitality of the neighborhoods are mostly considered, and the function of human habitation is considered as only a basic function of the historical remains. Most of the residents have moved out of the region in the current stage of development, which results in problems of "empty nest" and "aging" in the historical and cultural blocks. To revitalize historical and cultural blocks and enhance the perception of landscape value and behavioral intentions of historical and cultural blocks, it is necessary to start with improving people’s livelihoods, and gradually realize the return of culture through the economic prosperity of the region. In the process of renewal and development of the neighborhood, the participation of multiple subjects should be emphasized, together with the integration of advantageous regional resources, and surrounding synergistic development; it is important to improve the demographic structure and economic level, make changes to the current self-balancing reform mode, population evacuation and resettlement, land resource bundling and other issues that all need to be considered in an integrated manner outside the historical and cultural block. At the same time, it is essential to attract and encourage people to come to Qingdao to start their own business to move in, combined with government preferential policies, continuing with the historical pattern and characteristic functions of the old streets and alleys, and restore the historical memories. However, this research has some bias in the research data and results, because both the website comment search index and the sample size of the network questionnaire are limited, the geographical radiation breadth is insufficient, and some tourists are less accustomed to public platforms for publishing comments, so there are limitations in the data acquisition and collection.

2.The landscape value perception of historical and cultural blocks has a positive and significant effect on tourists’ behavioral intentions: educational and learning value perception > historical and cultural value perception > ecological landscape value perception > leisure and entertainment value perception. Landscape value perception of historical and cultural blocks has a positive and significant effect on local identity: educational and learning value perception > historical and cultural value perception > ecological landscape value perception > recreation value perception. This is consistent with the results of other scholars, such as Wei Mei [48] and Zhang Xi [49]. In this paper, we innovatively start from the landscape value perception of historical and cultural blocks to explore the relationship between landscape value perception and tourists’ behavioral intentions, to give researchers real feedback on the renewal and transformation of historical and cultural blocks as well as the research results of the strategy by means of the method of perceptual evaluation. Starting from the perspective of tourists’ perceptions and taking tourists’ behavioral intentions as the feedback result, the study embodies the influential relationship between the dimensions of landscape value perception in historical and cultural blocks, to provide a new direction for the renewal and development of historical and cultural blocks. The results of the study show that behavioral intentions are the feedback performance of the emotional connection between tourists and recreational areas, tourists’ perceptions of the value of skill and education and the value of history and culture have a stronger impact on tourists’ behavioral intentions, and maintain consistency with the local identity of tourists in the recreational areas. Through the analysis of the tourists’ comments, the text of emotional inclinations underlying the reasons can be understood. The majority of the content is concentrated on parking, catering and shopping, transportation, etc., which are related directly to the lack of supervision and management of the marketing of historical and cultural blocks and the lack of sound supporting facilities and infrastructure. In addition, the gradual ossification of the historical and cultural blocks is in line with the results of the research on the homogenization of the image of the tourist destinations of the ancient towns south of the Yangtze River, such as Chu Chit-hui [50], etc., and the lack of in-depth excavation of the differentiated characteristics of the historical and cultural blocks in the development of tourism. It is difficult for tourists to deeply understand the cultural characteristics of the neighborhoods in the horse-riding style of tourism. The problem of weak cultural perceptions of tourists also exists in other historical and cultural blocks [51, 52]; tourism development has sacrificed the historical features and tourists’ sense of place, and in the service facilities, the commodities are the same, and the commercialization has tended to become saturated, which means that the tourists’ sense of locality has been reduced [53]. Therefore, it is necessary to improve the service-oriented supporting facilities, prioritize pedestrianized systems, carry out precise planning and the refined management of on-street parking spaces, and strengthen the construction of parking guidance systems. The quality of the food service industry must be reduced in quantity and improved in quality, regional characteristics must be highlighted and market supervision must be strengthened. Through this study, we can provide positive feedback on the quality and renewal of historical and cultural blocks, relying on local characteristics and cultural resources, solving the problems in the dimensions of perceived value of recreation and entertainment, increasing the value perception of tourists, and realizing the comprehensive manifestation of the value of the neighborhoods and sustainable development.

3.The hypothesis of local identity as a mediating variable between the perception of ecological landscape value and the perception of educational and learning value is not maintained, and local identity has no mediating effect on the perception of ecological landscape value and the perception of educational and learning value. Local identity partially mediated the perceived value of history and culture and the perceived value of leisure and recreation. This may be related to the study site; the Qingdao historical and cultural block is the material carrier of the history of historical change, with strong historical and cultural attributes. However, the linkage of tourists’ behavioral intention does not need to be mediated by place identity, if we choose to take the natural ecological landscapes as the study site, such as Qingdao Badaguan Historical and Cultural block. Whether the ecological landscape value perception and the skill and educational value perception will significantly affect the tourists’ place identity and influence tourists’ behavioral intentions, needs to be further explored in future research.

4.This study explores the interplay between perceived landscape value and tourists’ behavioral intentions in historical and cultural blocks through qualitative analysis. By summarizing the dimensions of tourists’ perception of landscape value and using the quantitative path relationship of the structural equation model, the mechanism of the role of landscape value perception and tourists’ behavioral intention is revealed, thus providing real feedback for the renewal and renovation of historical and cultural blocks as well as for strategic research. This study not only enriches the theoretical research in the field of cultural tourism, but also provides guidance for practical tourism management and cultural preservation strategies, emphasizing the importance of systematic evaluation of landscape values from the perspective of tourists’ perceptions. In the theoretical aspect of the research: the innovative generalization of landscape value dimensions, through the qualitative research method, innovatively refining the landscape value perception dimensions into four aspects: ecological landscape, history and culture, skill education and recreation. Different from previous scholars from macro perspectives such as economics, management, aesthetics, etc., this study divides the dimensions of landscape value perception from the perspective of tourists, which provides a new perspective and research framework for the subsequent classification of landscape value perception, and promotes the academic community’s understanding of the diversity of landscape value. In terms of analyzing the relationship between behavioral intention and perceived landscape value: through the application of structural equation modeling, this study reveals the path coefficient relationship between tourists’ behavioral intention and perceived landscape value. This finding contributes to the understanding that tourists influence behavioral intention through different dimensions of landscape perception, which is an important contribution to supplementing the mechanism of the role between perceived value and behavioral intention. The conclusions of this study are also of practical significance: firstly, it proposes a strategy for the development of cultural tourism, and the enhancement of the perceived landscape value of historical and cultural blocks can significantly affect tourists’ behavioral intentions, which is of practical significance in guiding the development of management and marketing strategies for historical and cultural blocks. By improving service facilities and optimizing visitor experience, visitor satisfaction and sense of local identity can be effectively enhanced. On the other hand, the study emphasized that historical and cultural blocks should take into account the residents’ living aspirations and the preservation of history and culture when developing tourism functions. By maintaining the participation of multiple actors and emphasizing local characteristics in the renewal and development process, historic blocks can be effectively revitalized and their sustainable development promoted;

This study still has limitations: it only explains how tourists understand and perceive the visual elements of the landscape from a unidirectional perspective of landscape perception, i.e., it explores the influential mechanism of landscape elements on tourists’ behavioral intention from the perspective of the built environment of the neighborhood. However, the question of how to explain the image construction process of tourist destinations through "behavioral intention" from a multidirectional perspective needs to be further considered in subsequent studies. The findings of this study can improve the relevance of policy making and solve the problems of "empty nests" and "aging" in historical and cultural blocks through the integration of regional resources and preferential policies of the government, which provides a basis for local governments to formulate relevant cultural tourism and social policies. This provides a basis for local governments to formulate relevant cultural tourism and social policies, and helps to formulate more effective strategies to promote the dual development of regional economy and culture.

## 4. Conclusions

This study explores the interplay between perceived landscape value and tourists’ behavioral intentions in historical and cultural blocks through qualitative analysis. By summarizing the dimensions of tourists’ perception of landscape value and using the quantitative path relationship of the structural equation model, the mechanism of the role of landscape value perception and tourists’ behavioral intention is revealed, thus providing real feedback for the renewal and renovation of historical and cultural blocks as well as for strategic research. This study not only enriches the theoretical research in the field of cultural tourism, explore the multidimensional nature of perceived landscape value in historical and cultural blocks, but also provides guidance for practical tourism management and cultural preservation strategies, emphasizing the importance of systematic evaluation of landscape values from the perspective of tourists’ perceptions. In the theoretical aspect of the research: the innovative generalization of landscape value dimensions, through the qualitative research method, innovatively refining the landscape value perception dimensions into four aspects: ecological landscape, history and culture, skill education and recreation. Different from previous scholars from macro perspectives such as economics, management, aesthetics, etc., this study divides the dimensions of landscape value perception from the perspective of tourists, which provides a new perspective and research framework for the subsequent classification of landscape value perception, and promotes the academic community’s understanding of the diversity of landscape value. In terms of analyzing the relationship between behavioral intention and perceived landscape value: through the application of structural equation modeling, this study reveals the path coefficient relationship between tourists’ behavioral intention and perceived landscape value. This finding contributes to the understanding that tourists influence behavioral intention through different dimensions of landscape perception, which is an important contribution to supplementing the mechanism of the role between perceived value and behavioral intention. he conclusions of this study are also of practical significance: firstly, it proposes a strategy for the development of cultural tourism, and the enhancement of the perceived landscape value of historical and cultural blocks can significantly affect tourists’ behavioral intentions, which is of practical significance in guiding the development of management and marketing strategies for historical and cultural blocks. By improving service facilities and optimizing visitor experience, visitor satisfaction and sense of local identity can be effectively enhanced. On the other hand, the study emphasized that historical and cultural blocks should take into account the residents’ living aspirations and the preservation of history and culture when developing tourism functions. By maintaining the participation of multiple actors and emphasizing local characteristics in the renewal and development process, historic blocks can be effectively revitalized and their sustainable development promoted; The findings of this study can improve the relevance of policy making and solve the problems of "empty nests" and "aging" in historical and cultural blocks through the integration of regional resources and preferential policies of the government, which provides a basis for local governments to formulate relevant cultural tourism and social policies. This provides a basis for local governments to formulate relevant cultural tourism and social policies, and helps to formulate more effective strategies to promote the dual development of regional economy and culture.

## Supporting information

S1 File Data. (ZIP)
